# Determination of Sulfonamide Residues in Food by Micellar Liquid Chromatography

**DOI:** 10.1080/15376510701624043

**Published:** 2008-06-23

**Authors:** Arkadiusz Szymański

**Affiliations:** Faculty of Chemistry, Adam Mickiewicz University, Grunwaldzka 6 60-780, Poznań, Poland

**Keywords:** Food, Micellar Liquid Chromatography, Residue, Sulfonamides

## Abstract

This paper presents new methods of determination of sulfonamide residues in food products of animal origin, based on liquid chromatography with a micellar mobile phase. The methods employ a technique of direct injection of the sample and preliminary isolation of the analyte by extraction in the liquid-solid and liquid-liquid system. The methods have been characterized by providing the parameters of the calibration curves, the range of linearity, limit of detection, and precision and accuracy of particular determinations. The recovery of the sulfonamides introduced into the food products studied has also been determined.

## INTRODUCTION

In modern methods of animal production an increase is achieved among others by the application of pharmaceuticals not only for the treatment of animals, but also for stimulation of their growth and protection of their health. Along with the advantages, such procedures also have some drawbacks—trace amounts of the pharmaceutical substances are left in the food products obtained from such animals.

The drugs whose residues in food products are particularly undesirable are those containing sulfonamides. These drugs have antibacterial activity and are used for the treatment and for prophylactic purposes in animals, poultry, milk cows, fish, and even bees. Among these compounds, particular attention should be given to sulfamethazine suspected of carcinogenic effect (Littlefield 1988). Many states have described the admissible amounts of these pollutants in food products; in EU members the admissible level is 100 μg sulfonamides in 1 kg of food (Commission Regulation 1999).

One of the most often used methods for the analysis of sulfonamide residues in food products of animal origin is liquid chromatography (Agarwal 1992). When used with an UV-Vis detector, it does not require generation of derivatives of the compounds determined. The method presented in this paper is based on a high-pressure liquid chromatography with a micellar mobile phase.

## MICELLAR LIQUID CHROMATOGRAPHY

**FIGURE 1:**
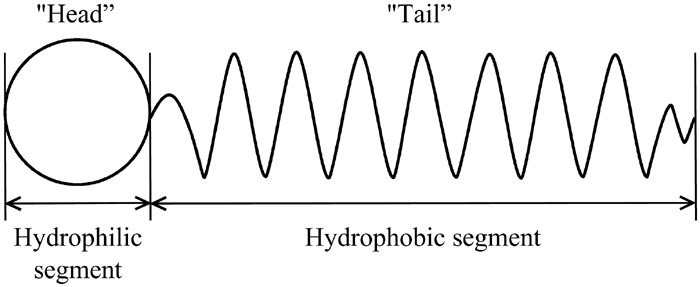
The basic molecular structure of a surfactant molecule.

The most important components of the mobile phase in micellar liquid chromatography are surfactants. They are characterized by a specific structure and properties determined by this structure. The main feature distinguishing this group of compounds is their amphiphilic nature resulting from the presence of a pronounced hydrophilic group and a nonpolar hydrophobic group in a molecule (Fig. 1).

Amphiphilic structure of these compounds determines their properties, of which the most important for chromatography is the surface activity, micelle formation, and ability to solubilize. At first surfactants were used in ionic-pair chromatography. Evolution of this technique has led to the use of surfactants in liquid chromatography in concentrations below the critical micellization concentration (cmc), which initiated development of the micellar liquid chromatography (MLC).

**FIGURE 2:**
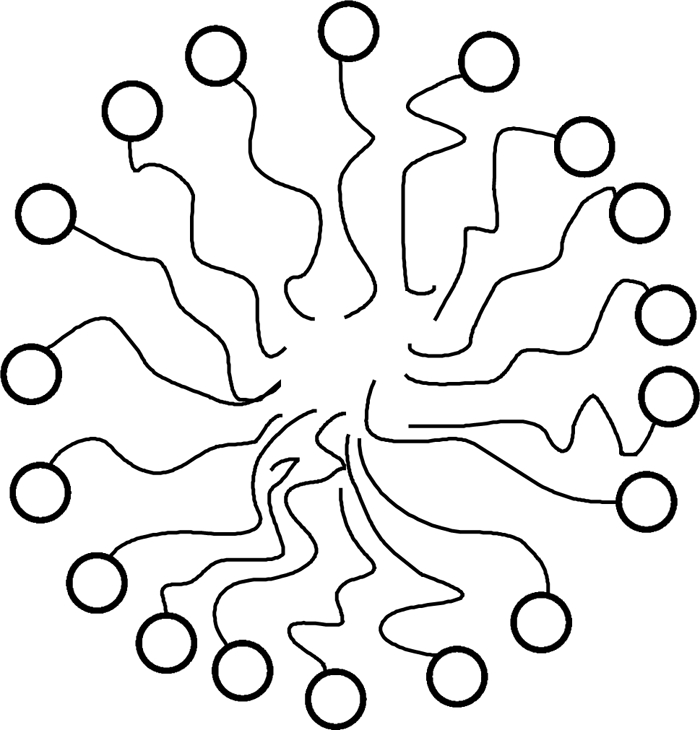
The normal micelle.

The distinguishing property of MLC is the composition of the mobile phase, which in microstructural level can be treated as heterogenic. Micellar mobile phase is usually a water solution of ionic or nonionic surfactants in a concentration above cmc, so besides the monomeric molecules the dominant forms in the solution are micelles (Fig. 2). Depending on the type of surfactant and properties of the mobile phase (concentration, ionic strength, temperature, pressure), the shape of micelles is different. Usually the ionic micelles have a spherical shape.

**FIGURE 3:**
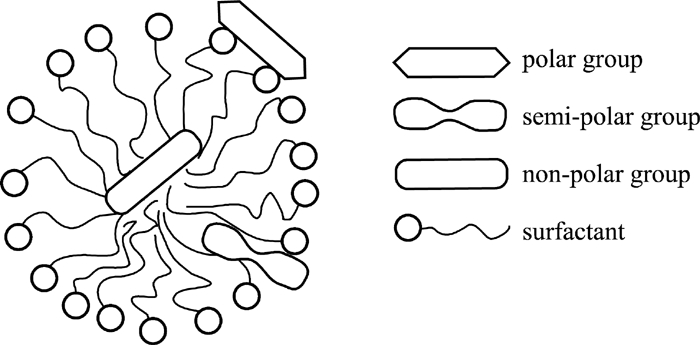
Solubilization.

Because of the specific interactions between micelles and a substance in water phase, the substance can be separated in the phenomenon called solubilization. The molecules of this substance can be adsorbed on the surface or inside of the micelle. The phenomenon of solubilization is illustrated in Figure 3.

Many authors have been interested in the applications of MLC (Szczepaniak and Szymański 1997). The main advantages of this method include unique selectivity related to the interactions between the micelle and the chromatographed substance, a possibility of separation of the ionic and nonionic substances on the same filling, low cost of the mobile phase, nontoxicity and inflammability of the mobile phase, and possibility of determination of the biologically active substances in body fluids (blood serum, urine) by a direct sample injection technique without preliminary separation of the analyte. Thanks to the unique properties of the micellar phase, the biological matrices, and in particular proteins, undergo solubilization, permitting a determination of drugs in blood serum (Szczepaniak and Szymański 1995).

The aim of the study reported was to work out methods for determination of the sulfonamide residues in food products of animal origin taking advantage of the MLC technique.

## EXPERIMENTAL

### Reagents and Apparatus

#### Surfactant

Anionic surfactant for making micellar mobile phases was sodium dodecyl sulfate (SDS) (Aldrich).

#### Sulfonamides

Sulfacetamide, sulfanilamide, sulfaproxyline, sulfamethazine, sulfadiazine, sulfachloropyridazine, sulfamerazine, sulfaguanidine, sulfadimethoxine, sulfathiocarbamide, sulfafurazole, sulfisomidine, sulfaquinoxaline, and sulfathiazole were from Sigma-Aldrich. Standard solutions of 1 g/L concentration were made in MeOH. Working solutions were obtained by subsequent dilutions with the mobile phase used.

#### Deionized Water

Deionized water for making mobile phases and standard solutions was obtained in Milli-Q (Millipore).

#### HPLC

Chromatographic study was performed on a chromatograph HP-1050 (Hewlett Packard) equipped with a UV-Vis detector (190–600 nm), an injector Rheodyne 7125 with 20 μL of the sample loop, and LiChrospher 100 RP-18e 5 μm column, 250 mm × 4,6 mm (Merck). To maintain constant temperature of MLC separations, a water thermostating system was used.

### Repeatability

Repeatability of the chromatographic system was tested by the injection of a pattern solution containing 1 μg/mL sulfamethazine (n = 9). Repeatability of the retention coefficient and peak height for analyte was 2.86 ± 0.008, RSD = 0.38%, and 9.75 ± 0.15, RSD = 0.11%. Even if the samples were injected manually, the obtained result, including RSD values, were quite repeatable and, thus, of good quality.

### Procedures

#### Preparation of the Micellar Mobile Phase

The mobile phase was obtained by dilution of a water solution of 0.2 M SDS with deionized water to about 950 mL and addition of appropriate amount of an organic modifier and orthophosphoric acid. The value of pH was adjusted by solid sodium hydroxide. After supplementing the amount of the phase with deionized water to 1000 mL, it was filtered through a 0.45-μm filter of cellulose acetate.

#### Extraction of Sulfonamides to Solid Phase

##### Cyclobond I Column Conditioning

Cartridges with sorbent were placed on a vacuum manifold, conditioned successively with 5 mL MeOH/H_2_O (1:1), 5 mL of deionized water, and 5 mL of phosphate buffer of pH 5.1, not allowing the bed to dry.

##### Sample Introduction

The sample extract to be analyzed (honey or milk) was filtered through the column at a rate of 1 to 3 mL/min. The sorbing was washed with 2 mL of deionized water and dried in a vacuum system for 20 min.

##### Elution

The analyte was washed out with 2 × 5 ml MeOH and evaporated, and the dry residue was dissolved in 1 mL of mobile phase.

#### Preparation of a Sample of Honey for Analysis

##### Direct Injection

Five measuring flasks of 10 mL in capacity were loaded with 4.00 g of honey and 0, 250, 500, 750, and 1000 μL of the standard solution containing sulfadiazine, sulfamerazine, sulfathiazole, and sulfaguanidine; each compound was in the amount of 10 μg/1 mL of the solution, and then the flask was supplemented with mobile phase. The solutions were filtered through a filter of 0.45 μm made of cellulose acetate and then injected onto the column.

##### Solid Phase Extraction

Five flasks of 25 mL in capacity were loaded with 1 g of honey, then with the standard solutions prepared as above and supplemented with phosphate buffer of pH 5.0. The column with Cyclobond I sorbent was conditioned with 5 mL of deionized water and 5 mL of phosphate buffer of pH 5.1. The dissolved samples of honey were filtered through the column and washed with 2 mL of deionized water. The bed was dried in air under reduced pressure and the sulfonamides retained were eluted with 10 mL MeOH. The solvent was evaporated on a vacuum evaporator at a temperature below 40°C, and the residue was supplemented with the mobile phase to 1 mL and subjected to chromatographic analysis.

##### Liquid-Liquid Extraction

A portion of 20 g of honey was dissolved in 10 mL of phosphate buffer of pH 5.3 and transferred to a separator of 125 mL in capacity. Portions of 0, 50, 200, and 500 μL of sulfathiazole and sulfamerazine in a concentration of 10 μg/mL in phosphate buffer were added and the contents were twice shaken for 15 min with a mixture of acetone/chloroform at the ratio 2:1. The combined organic extracts were evaporated and the dry residue was dissolved in 1 mL of mobile phase.

#### Preparation of Milk and Egg Samples for Analysis

##### Direct Injection

Milk sample to be analyzed is passed through a filter 0.45 μm made of cellulose acetate and directly injected onto the chromatographic column.

##### Calibration Curves

Six 10-mL flasks were loaded with known amounts of the sulfonamides studied and the flasks were supplemented with milk to get solutions of each sulfonamide in concentrations from 0.25 to 5.0 μg in 1 mL of milk. Such solutions were subjected to chromatographic analysis.

##### Solid Phase Extraction and Liquid-Liquid Extraction

A sample of milk (10 mL) or eggs (3 g) was prepared as follows. In order to remove proteins the sample was twice treated with 25 mL of 3% trichloroacetic acid, shaken for 20 min, and filtered. The pH of the combined solutions was adjusted by NaOH to be 5.1. The sample was analyzed.

##### SPE

A column of 3 mL in capacity was filled with 500 mg of Cyclobond I, which was conditioned with 5 mL of a 1:1 solution of methanol and water, 5 mL of water, and then 5 mL of phosphate buffer of pH 5.1. The solution to be analyzed was passed through such prepared bed taking care not to air the column packing. Then 2 mL of water was passed through the column in order to remove the phosphates. The sulfonamides retained on the solid phase were washed out with 10 mL of methanol. After the solvent evaporation in a vacuum evaporator the dry residue was dissolved in 1 mL of the mobile phase and subjected to chromatographic analysis.

##### LLE

After removal of proteins and pH adjustment, the solution was subjected to extraction twice from 25 mL of methylene chloride. The combined organic extracts were evaporated in a vacuum evaporator, and the dry residue was dissolved in 1 mL of the mobile phase and then it was subjected to HPLC analysis.

##### Calibration Curves

Six conical flasks of 50 mL in capacity were charged with 10 mL of milk or 3 g of an egg, and with known amounts of the sulfonamides studied to get solutions of concentrations from 0.2 to 5.0 μg of each sulfonamide in 1 mL of milk or in 1 g of homogenized egg. The samples were subjected to the HPLC analysis.

#### Preparation of Meat and Blood Serum Samples

##### Preparation of a Meat Sample

Carefully weighted 5.0 g of refined poultry meat was homogenized for 5 min with 25 mL of chloroform and centrifuged for 10 min at 3000 rot/min. The liquid was decanted and filtered through a filter made of cellulose acetate and the solid residue was re-extracted with 25 mL of chloroform. The filtrates were combined and evaporated in a vacuum evaporator at 40°C, and the residue was dissolved in 1 mL of the mobile phase. The solution was filtrated through a membrane filter of pore size 0.45 μm and then injected onto the chromatograph.

##### Preparation of a Blood Serum Sample

Carefully measured 10 mL of bovine blood serum was shaken with 20 mL of 3% trichloroacetic acid for 10 min, and then the sample was centrifuged at 4000 rot/min and decanted. The pH value of the solution was adjusted to 5.1 by adding solid sodium hydroxide, and then 50 mL of chloroform was added and the mixture was extracted for 10 min with chloroform. The extract was evaporated in a vacuum evaporator at 40°C. The dry residue was dissolved in 1 mL of the mobile phase, filtered, and injected onto the LC.

##### Calibration Curve Determination

The calibration curves for measurements of sulfadiazine, sulfamethazine, and sulfadimethoxine were made on the basis of the chromatographic analysis of meat and blood serum samples dotted with the standard solutions of the sulfonamides studied. The samples used for calibration contained 0.05, 0.1, 0.25, 0.5, 1.0, 2.0, and 4.0 μg of each sulfonamide in 1 g of meat and 0.05, 0.1, 0.25, 0.5, 1.0, 1.5, and 2.0 μg in 1 mL of blood serum. Two parallel samples were made.

##### Sample Preparation for Determination of Recovery

Having confirmed the absence of sulfonamides studied in the samples analyzed, on the basis of these samples we prepared from the samples containing known amounts of these compounds. To 5.0 g of meat the amounts of 250 μL or 125 μL of the standard solution containing the sulfonamides in the concentration of 10 μg/mL were added. Thus, the samples contained 0.5 μg and 0.25 μg of sulfonamides in 1.0 g of meat. Into 10 mL of the analyte being the blood serum the amounts of 500 μL or 200 μL of the standard solution were introduced, so that the blood serum analyzed contained 0.5 μg/mL and 0.2 μg/mL of the sulfonamides studied. In each case, according to the procedure, five samples were measured.

## RESULTS AND DISCUSSION

### Mechanism of Sulfonamides Retention in MLC

At the first stage the behavior of sulfonamides in MLC was examined. The influence of the surfactant concentration in the mobile phase, pH of the mobile phase, concentration and type of the organic modifier, temperature of the column, and type of the stationary phase on the sulfonamide retention was determined (Szczepaniak and Szymański 1998). Results of these preliminary determinations permitted a final choice of the stationary phase, temperature of separation, and type of organic modifier and the preliminary choice of parameters of the mobile phase used in particular analysis. The final parameters of the eluent (surfactant concentration and pH of the mobile phase) depended on particular sulfonamides and the matrices studied.

As sulfonamides are of amphoteric character, the main parameter influencing the retention and permitting optimization of the separating system is the pH of the mobile phase. The acidic character is related to the electron accepting properties of the substituent R at the sulfonamide group. The scheme presents the equations of protonation of the amine group and dissociation, that is, abstraction of the proton at the nitrogen atom in the sulfonamide group. Table 1 presents structures of the sulfonamides that can occur in the preparations of animal origin whose residues are determined in food products.

**TABLE 1:** Structures of the sulfonamides studied

R		R	
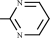	Sulfadiazine	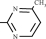	Sulfamerazine
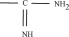	Sulfaguanidine	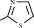	Sulfathiazole
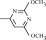	Sulfadimethoxine	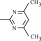	Sulfamethazine
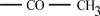	Sulfacetamide		

The aim of the optimization was to obtain full separation of the sulfonamides studied in a possibly short time at a simultaneous elimination of possible interferences of the peaks assigned to the analyte and the matrices.

**FIGURE 4:**
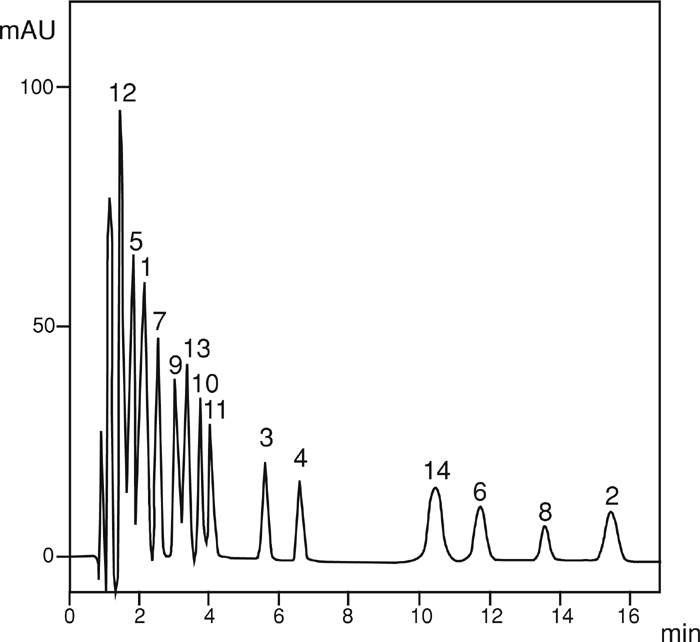
Chromatogram MLC of 14 sulfonamides. The mobile phase: SDS = 0.04 M; pH = 4.6; 2-Pr-OH = 2%; [PO_4_] = 0.01 M; T = 40°C; flow rate, 1 mL/min. Peaks: 1. sulfathiocarbamide, 2. sulfaproxyline, 3. sulfachloropyridazine, 4. sulfafurazole, 5. sulfacetamide, 6. sulfadimethoxine, 7. sulfadiazine, 8. sulfisomidine, 9. sulfamerazine, 10. sulfathiazole, 11. sulfamethazine, 12. sulfanilamide, 13. sulfaguanidine, 14. sulfaquinoxaline.

Chromatographic separation was performed in the isocratic conditions on a column filled with C-18. The micellar mobile phase was a water solution of 0.02 to 0.1 M SDS (sodium dodecyl sulfate) of cmc = 8.1 × 10^−3^ M, 0.01 M phosphate buffer and 1% to 2% addition of 2-propanol used as an organic modifier. Figure 4 presents the exemplary separation of 14 sulfonamides.

At the next step the procedures for determination of sulfonamide residues in milk, poultry meat, bovine blood serum, milk, and eggs were proposed. The most important task was to prepare samples for analysis. For honey, milk, and blood serum the preparation was limited to filtering or dissolving the sample as the MLC method permitted direct injection of these samples. The sulfonamide residues were always isolated from the natural matrices and concentrated by the SPE and LLC methods. Isolation of the analyte is the preliminary and essential condition for getting a reliable final result. For determination of each sulfonamide in each type of sample the linear range of the calibration curve and the correlation coefficients were determined together with the detection limit, precision, and accuracy of the method.

The detection limit was calculated for the analyte concentration giving a signal three times as high as the mean noise recorded for the matrix that did not contain the sulfonamide determined. The precision of determinations was described by R.S.D., while the accuracy of the method was measured by the recovery of the sulfonamides additionally introduced into the solutions analyzed.

### Determination of Sulfonamide Residues in Food Products

#### Analysis of Sulfonamides in Honey

The sulfonamides most often occurring in the preparations used in bee raising are sulfamerazine, sulfathiazole, sulfaguanidine, and sulfadiazine. These compounds were determined in honey samples by the method proposed (Szymański 2000). The calibration curves in the linearity region are characterized by a high correlation coefficient. The limit of detection of the above mentioned sulfonamides is 0.1 μg/g honey.

**FIGURE 5:**
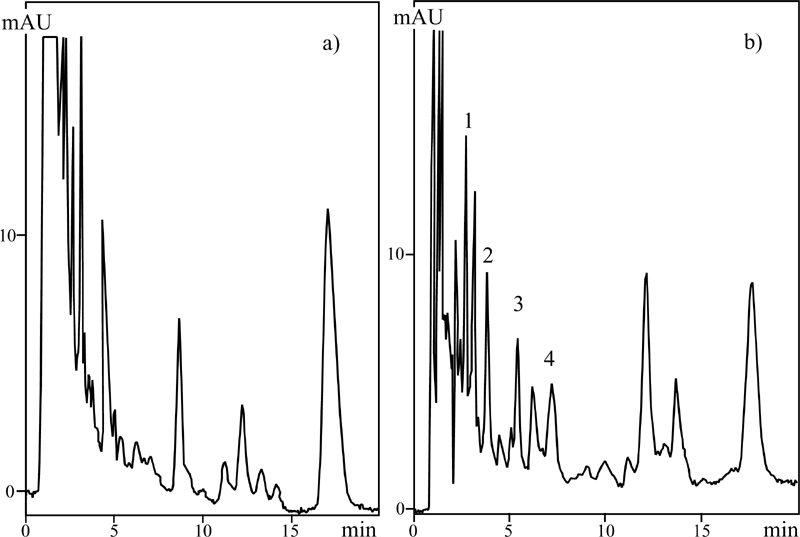
MLC (DSI) chromatograms of (a) sample of honey, (b) sample of honey with additionally introduced sulfonamides in the amount 0.25 μg/g; mobile phase: SDS = 0.04 M; pH = 3.5; 2-PrOH = 2%; [PO_4_] = 0.01 M; T = 40°C. Peaks: 1. sulfadiazine, 2. sulfamerazine, 3. sulfathiazole, 4. sulfaguanidine.

When applying the liquid-liquid extraction as the concentration technique, it proved impossible to determine a few sulfonamides simultaneously as other compounds present in honey were concentrated together with the sulfonamides. This method was used to determine sulfathiazole and sulfamerazine reaching the detection limit of 0.02 μg/g honey; precision of 3.8% and 2.8% for sulfathiazole and sulfamerazine, respectively, in the samples with additionally introduced sulfonamides in the amount of 0.05 μg/g; and 2% accuracy for both sulfonamides (Table 2). Figure 5 presents results of chromatographic separations used for determination of sulfonamides in honey with direct sample injection.

**TABLE 2:** Statistical analysis of sulfonamide determination in honey (n = 5)

Sulfonamide	Added (μg/g)	Found (μg/g) (± SD)	Recovery (%)	R.S.D. (%)	Accuracy (%)
DSI					
Sulfamerazine	1.25	1.26 ± 0.02	100.8	4.20	0.8
Sulfathiazole	1.25	1.26 ± 0,04	100.8	6.99	0.8
Sulfaguanidine	1.25	1.25 ± 0,08	100.0	3.82	0.0
SPE					
Sulfadiazine	0.50	0.50 ± 0,01	100.0	2.83	0.0
Sulfamerazine	0.50	0.50 ± 0,01	100.6	1.90	0.0
Sulfathiazole	0.50	0.51 ± 0,01	102.0	4.23	2.0
Sulfaguanidine	0.50	0.49 ± 0,09	97.0	3.57	2.0
LLE					
Sulfamerazine	0.05	0.051 ± 0.002	102.0	2.8	2.0
Sulfathiazole	0.05	0.051 ± 0.006	102.0	3.9	2.0

#### Analysis of Sulfonamides in Poultry Meat and Bovine Blood Serum

The preparation procedure was relatively simple: carefully weighted refined meat (carefully measured bovine blood serum volume) was homogenized with 25 mL of chloroform and subjected to centrifuging (Szymański 2002). The liquid was decanted and filtered, and the solid residue was once again extracted with chloroform. The filtered extracts were combined and evaporated on a vacuum evaporator, the residue was dissolved in 1 mL of the mobile phase, and after filtration it was injected onto the chromatograph.

The limit of detection was 0.025 μg of sulfadiazine and sulfamethazine, and 0.05 μg of sulfadimethoxine in 1 g of meat (1 mL of bovine blood serum).

**FIGURE 6:**
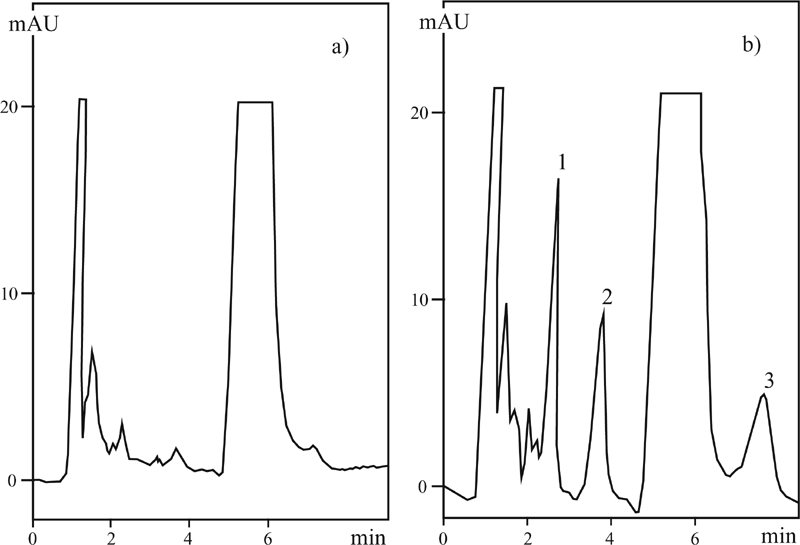
MLC chromatograms of (a) extract of meat without sulfonamides, (b) extract of meat with sulfonamides added in the amount of 0.25 μg/g; mobile phase: SDS = 0.04 M; pH = 2.8; 2-PrOH = 2%; [PO4] = 0.01 M; T = 40°C. Peaks: 1. sulfadiazine, 2. sulfamethazine, 3. sulfadimethoxine.

Figure 6 presents exemplary chromatographs of the sulfonamides determined, extract of the meat, and extract of the meat with additionally introduced sulfonamides. Full separation of the compounds determined was completed in 10 min.

The results (Table 3) prove that the method of simultaneous determination of three sulfonamides in meat and blood serum proposed in this work is characterized by high precision and accuracy and the recovery values obtained confirm its suitability for the purpose.

**TABLE 3:** Statistical analysis of determination of sulfonamides in poultry meat and bovine blood serum (n = 5)

*Meat*	Added (μg/g)	Found (μg/g) (± SD)	Recovery (%)	Accuracy (%)	R.S.D. (%)
Sulfadiazine	0.500	0.497 ± 0.011	99.4	0.6	4.26
	0.250	0.234 ± 0.004	93.6	6.4	3.67
Sulfamethazine	0.500	0.515 ± 0.014	103.0	−3.0	5.25
	0.250	0.241 ± 0.007	96.4	3.6	6.00
Sulfadimethoxine	0.500	0.487 ± 0.013	97.4	2.6	5.35
	0.250	0.218 ± 0.006	87.2	12.8	5.91
*Blood serum*	(μg/mL)	(μg/mL) (± SD)	(%)	(%)	(%)

Sulfadiazine	0.500	0.488 ± 0.021	97.7	2.4	6.60
	0.200	0.189 ± 0.003	94.5	5.5	3.74
Sulfamethazine	0.500	0.498 ± 0.021	99.6	0.4	6.28
	0.200	0.186 ± 0.003	93.0	7.0	4.04
Sulfadimethoxine	0.500	0.485 ± 0.089	97.0	3.0	4.12
	0.200	0.184 ± 0.004	92.0	8.7	4.86

### Determination of the Sulfonamide Residues in Milk and Eggs

**FIGURE 7:**
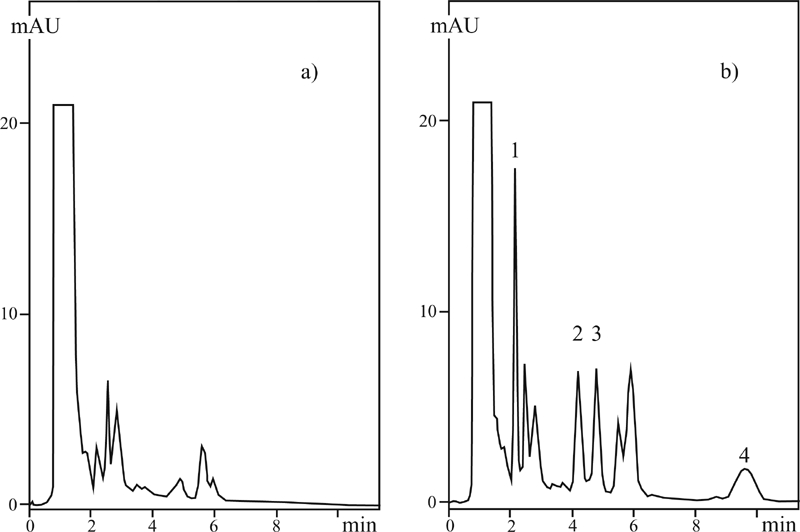
MLC (DSI) chromatograms of (a) milk without sulfonamides, (b) milk with sulfonamides introduced in the amount of 0.25 μg/g; mobile phase: SDS = 0.04 M; pH = 3.5; 2-PrOH = 2%; [PO_4_] = 0.01 M; T = 40°C. Peaks: 1. sulfacetamide, 2. sulfamethazine, 3. sulfathiazole, 4. sulfadimethoxine.

Figure 7 presents exemplary chromatograms permitting a determination of sulfonamide residues in milk obtained by the direct injection technique (Szymański 2003). Results of the analyses and statistical analysis are given in Tables 4 (milk) and 5 (eggs).

**TABLE 4:** Statistical analysis of sulfonamide determination in milk (n = 5)

	Added	Found	Recovery	Accuracy	R.S.D.
DSI	(μg/mL)	(μg/mL)	(%)	(%)	(%)
Sulfacetamide	0.10	0.105	105	5	7.1
	0.50	0.51	102	2	6.2
Sulfamethazine	0.10	0.103	103	3	5.5
	0.50	0.51	102	2	4.9
Sulfathiazole	0.10	0.104	104	4	5.3
	0.50	0.51	102	2	4.8
Sulfadimethoxine	0.10	0.105	105	5	4.8
	0.50	0.52	102	4	3.8
SPE	(μg/mL)	(μg/mL)	(%)	(%)	(%)

Sulfacetamide	0.05	0.051	102	2	5.0
	1.00	1.01	104	1	4.3
Sulfamethazine	0.05	0.052	104	4	4.2
	1.00	1.01	101	1	3.9
Sulfathiazole	0.05	0.051	102	2	5.3
	1.00	1.03	103	3	5.5
Sulfadimethoxine	0.05	0.052	104	4	8.0
	1.00	1.03	103	3	6.7
LLE	μg/mL	μg/mL	(%)	(%)	(%)

Sulfacetamide	0.05	0.051	102	2	4.2
	0.50	0.51	102	2	4.0
Sulfamethazine	0.05	0.049	98	−2	3.9
	0.50	0.49	98	−2	4.1
Sulfathiazole	0.05	0.051	102	2	4.8
	0.50	0.50	100	0	4.0
Sulfadimethoxine	0.05	0.052	104	4	3.5
	0.50	0.49	98	−2	2.0

**TABLE 5:** Statistical analysis of determination of sulfonamides in eggs (n = 5)

	SPE			LLE				
Sulfonamide	1	2	3	4	1	2	3	4
Added (μg/mL)	0.05	0.05	0.05	0.05	0.05	0.05	0.05	0.05
	0.40	0.40	0.40	0.40	1.00	1.00	1.00	1.00
Found (μg/mL)	0.049	0.051	0.049	0.048	0.049	0.049	0.051	0.048
	0.40	0.40	0.40	0.40	1.01	1.01	1.00	1.03
Recovery (%)	98	102	98	96	98	98	102	96
	100	100	100	100	101	101	100	103
R.S.D. (%)	4.2	5.3	5.1	3.4	8.0	5.7	6.6	3.5
	2.9	4.3	3.5	3.1	7.7	4.3	6.5	3.2
Accuracy (%)	−2	2	−2	−4	−2	−2	2	−4
	0	0	0	0	1	1	0	3

The method is characterized by high accuracy and precision; R.S.D. value does not exceed 8% for milk and eggs, and the accuracy varies from −8 to 5. The recovery of sulfonamides introduced into the samples of milk and eggs varies in the range of 92% to 105%.

The limit of detection achieved, collected in Table 6, depends on the method of preliminary sample preparation.

**TABLE 6:** Limits of detection (μg/kg)

	Honey	Meat	Serum	Milk	Eggs
DSI	250	—	—	100	—
SPE	250	—	—	50	25
LLE	20	25	25	25	25

## CONCLUSIONS

The methods proposed are suitable for determination of the sulfonamide residues in food products of animal origin. They permit analysis of the accuracy below the admissible level of sulfonamides in food. The methods proposed are characterized by high precision and accuracy. The application of the micellar mobile phase permits a direct injection of milk, honey, and physiological fluids, and if the products studied are meat or eggs, the method does not require a total elimination of protein and fat, which substantially simplifies the sample preparation procedure and shortens the analysis.
